# Future Protein Sources in Sports Nutrition: Sustainable Solutions​

**DOI:** 10.1007/s13668-026-00734-8

**Published:** 2026-02-16

**Authors:** Tuğba Tuna, Nesli Ersoy

**Affiliations:** 1 Faculty of Health Sciences, Department of Nutrition and Dietetics, Mudanya University, Bursa, Türkiye; 2 Faculty of Health Sciences, Department of Nutrition and Dietetics, Hacettepe University, Ankara, Türkiye

**Keywords:** Alternative protein sources, Sustainable nutrition, Sports nutrition, Environmental impact

## Abstract

**Purpose of Review:**

This review evaluates the potential of alternative protein sources, including plant-based proteins, insect proteins, mycoproteins, microalgae, and cultured meat, in meeting the increasing demand for sustainable and functional protein solutions in sports nutrition.

**Recent Findings:**

Studies indicate that alternative protein sources provide significant environmental benefits, such as reduced greenhouse gas emissions and lower water use, while delivering essential nutrients to support athletic performance and recovery. Advances in technology and production methods have further enhanced their feasibility as replacements for conventional animal-based proteins.

**Summary:**

Integrating alternative protein sources into sports nutrition offers a dual benefit: meeting athletes’ dietary requirements and supporting global sustainability goals. These protein sources pave the way for innovative, environmentally conscious dietary practices in sports nutrition, marking a pivotal shift toward a more sustainable future.

## Introduction

In recent years, the depletion of natural resources and rapidly growing population have become one of the most critical global challenges [32, 50]. The world population is projected to reach 9.8 billion by 2050, which will substantially increase the demand for macro- and micronutrients [18, 43, 75]. This demand is particularly pronounced for protein, as consumption patterns still rely heavily on animal-based sources. Therefore, ensuring a sustainable protein supply has emerged as a major concern for global food security [21, 57]. Proteins are essential for metabolic processes and play a critical role in growth and repair. Recent evidence highlights the strong connection between nutrition, health, and the environment; given the current limits of our planet, this underscores the urgent need for a comprehensive transformation of global food systems [23, 25].

Current food systems are the most significant contributors to environmental problems such as global greenhouse gas emissions, deforestation, and water consumption [31, 62]. Compared to plant foods, meat production causes significantly higher environmental damage, particularly in terms of greenhouse gas emissions and water use. For example, beef production generates 14–32 kg CO₂-eq per kilogram of meat in OECD countries, while pork and poultry emit 3.9–10 kg and 3.7–6.9 kg CO₂-eq, respectively. Globally, beef accounts for about 41% of livestock-related GHG emissions and is responsible for approximately 44% of total methane and 53% of nitrous oxide emissions from animal agriculture, corresponding to 7.1 gigatonnes CO₂-eq annually [67].

In contrast, many plant-based meat analogues, such as soy- or pea-based products, emit between 0.3 and 4.0 kg CO₂-eq/kg [79] with notable production-stage differences; for instance, oats and lentils have cultivation emissions of ~ 1.3 kg CO₂-eq/kg, while beans have only ~ 0.1 kg CO₂-eq/kg [70]. Furthermore, soy provides relatively low carbon emissions but requires 2.4 m³/kg of water, whereas peas have low emissions and water use but face production limitations due to climate constraints [70]. These impacts collectively underscore the substantial role of livestock in accelerating global warming, given that protein production from ruminants such as beef and lamb causes approximately 250 times more emissions than legumes, and ruminant meats produce significantly higher greenhouse gases than poultry [22, 82].

Alternatives to existing protein sources are needed to realize the change toward sustainable and healthy nutrition. The demand for products with a low environmental impact is increasing, especially among individuals seeking high-quality protein intake, such as athletes. Nutritional science and technology research has focused on searching for and utilizing alternative protein sources that provide adequate human nutrition and have a lower environmental impact [23, 25]. Therefore, it is essential to search, investigate, and implement new protein sources that have not been used so far or to improve existing ones. These data have led to the examination of new protein sources that can be used in products, especially for athletes in the future. At the same time, these proteins are expected to solve the problems of balanced nutrition in the future, at least partially. Alternative protein sources such as plant-based substitutes, cultured meat, and insects represent potential solutions that can positively affect our planet and human population, both in developing countries with food security concerns and in developed countries with environmental and animal welfare problems [87].

Adequate protein intake is not only critical for general health but also plays a fundamental role in sports nutrition, where it supports training adaptation, muscle repair, and recovery [2, 92]. Athletes generally require higher protein intakes than the general population, with consensus statements from the International Society of Sports Nutrition (ISSN) and the American College of Sports Medicine (ACSM) recommending 1.4–2.0 g/kg/day depending on training type and intensity. Endurance athletes may benefit from 1.2 to 1.6 g/kg/day, while strength or power athletes often require intakes closer to 1.6–2.0 g/kg/day to optimize lean mass and repair [34, 37]. Elevated protein needs may also arise during injury, energy restriction, or heavy training periods [83]. This increased demand, along with the growing trend among athletes to use supplements for enhancing performance and recovery [52], has driven interest in sustainable protein alternatives. As the global sports nutrition market continues to expand [7, 64], the development of environmentally friendly, high-quality protein sources has become essential to align athletic needs with planetary health goals [4, 60].

### Alternative Protein Sources in Sustainable Sports Nutrition

**Fig. 1:**
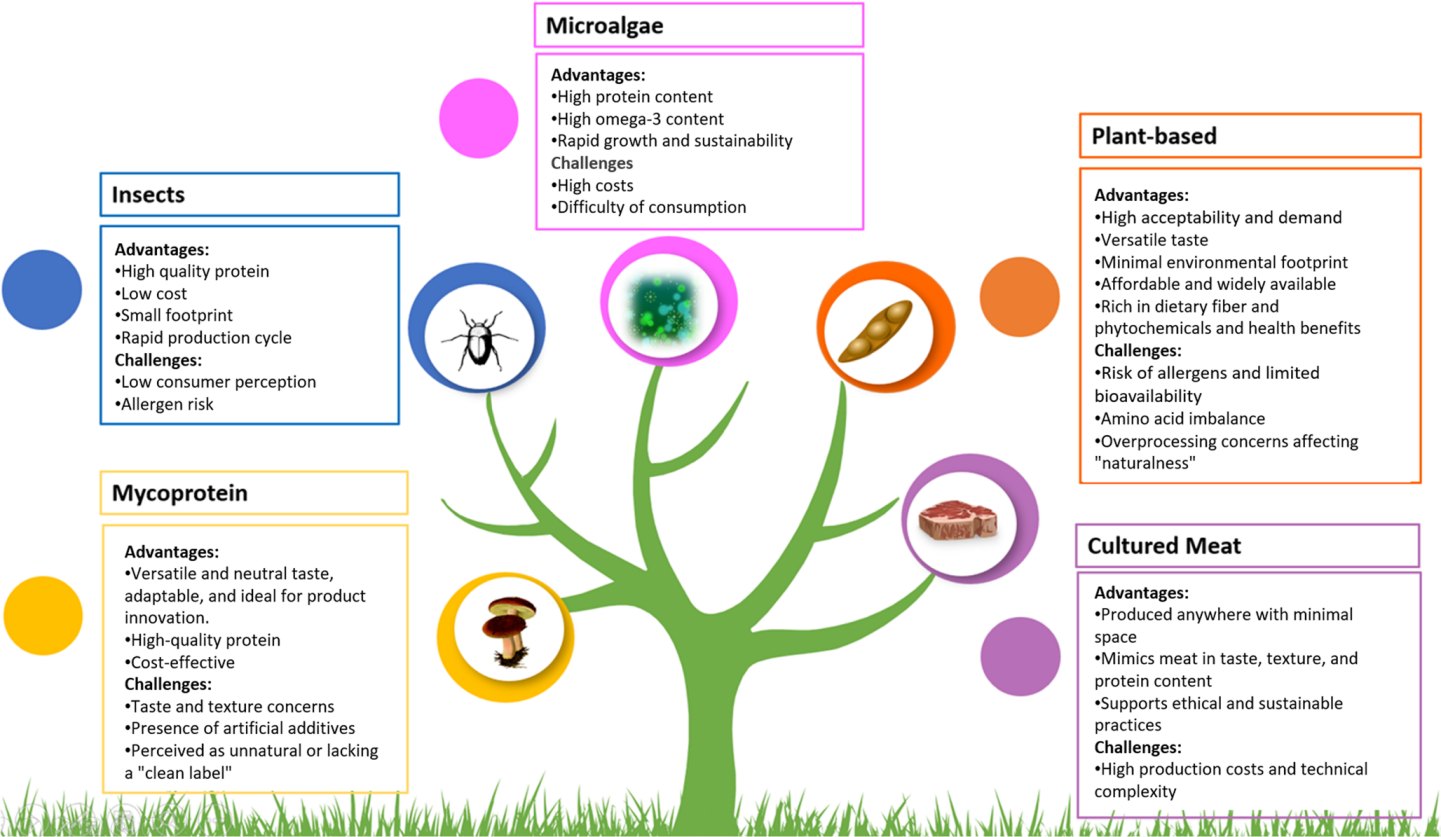
Advantages and challenges of alternative protein sources *(created by the authors based on *[17, 61, 71, 86])

Alternative protein sources are gaining increasing attention as part of global strategies to build sustainable food systems, particularly within the framework of the Sustainable Development Goals 2030 [15, 89]. Among these, plant-based proteins are highly acceptable and widely available, with a versatile taste and minimal environmental footprint, though concerns remain regarding amino acid imbalance and allergenicity [61]. Insects provide high-quality protein with low production costs and a small ecological footprint, but consumer perception and allergenicity remain barriers [86]. Mycoprotein is another promising option due to its adaptability and protein density, although it is sometimes perceived as unnatural or lacking a “clean label” [86]. Microalgae contribute not only high protein content but also valuable omega-3 fatty acids and rapid growth potential, although production costs are still high [17]. Finally, cultured meat (lab-grown meat) mimics conventional meat in taste, texture, and protein quality and supports ethical and sustainable practices, but its scalability remains limited by high costs and technical complexity [61, 71]. These advantages and challenges of alternative protein sources are summarized in Fig. 1.

#### Plant-Based Protein

**Fig. 2:**
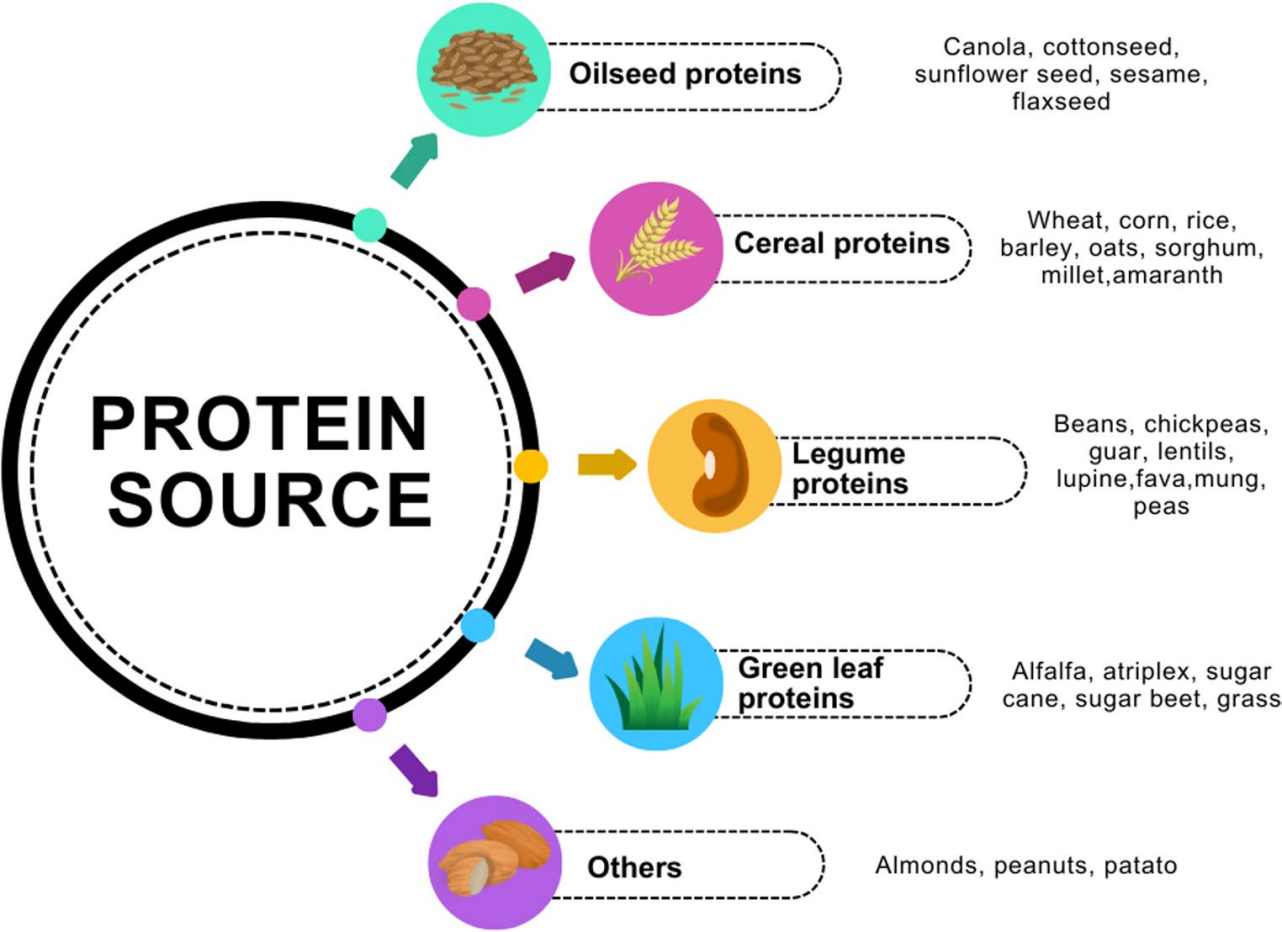
Main plant protein sources used in sustainable food production and sports nutrition *(created by the authors based on* [6, 40, 77])

Plant proteins are increasingly being used as alternatives to animal-derived proteins in human nutrition and as functional ingredients in food production [63, 68]. Rising costs and the limited supply of animal proteins—driven by climate change, freshwater depletion, and health risks—have accelerated the shift toward plant-based sources. Legumes, cereals, pseudocereals, seeds, almonds, and hazelnuts are commonly used as protein supplements. However, some plant proteins are considered less nutritionally complete compared to animal proteins [74]. Soy protein, in particular, remains one of the most widely used meat analogs due to its favorable properties and affordability. In addition, proteins obtained from oilseeds and fermentation-based processes are also used in meat analogs. Industrial efforts increasingly focus on protein-rich materials such as wheat, rice, corn, oilseeds, cereals, and legumes (Fig. 2) [6, 40, 77].

Although plant proteins generally have lower protein quality compared to animal proteins, they offer additional benefits that make them attractive in sports nutrition. Diets rich in vegetables are associated with reduced oxidative stress and inflammation [5]. Oxidative stress, caused by an imbalance between reactive oxygen species and antioxidant defenses, is linked to cellular damage and impaired performance [47]. While moderate exercise is beneficial, acute bouts of high-intensity exercise can exacerbate oxidative stress, leading to muscle fragility. In this context, antioxidant-rich plant proteins are valuable: polyphenols and other bioactive compounds may reduce inflammation, mitigate oxidative stress, and support athletic performance [8]. Therefore, plant proteins may serve as ergogenic aids, sustainable options, and value-added alternatives in sports nutrition.

Several clinical trials and reviews have investigated the effects of plant proteins on muscle health and performance. Lynch et al. [49] demonstrated that soy and whey protein similarly supported strength and lean mass gains during resistance training when leucine levels were equalized. Röhling et al. [72] found that soy protein supplementation reduced exercise-induced muscle stress during endurance training, while Jin et al. [35] showed that oligopeptide mixtures (soy, wheat, fish collagen) improved post-exercise lipid metabolism in cyclists. A recent review emphasized that with adequate amounts and complementary sources, plant proteins can support muscle protein synthesis and strength performance comparable to animal proteins. Likewise, a meta-analysis reported that plant-based diets do not compromise muscular strength compared to omnivorous diets, reinforcing their compatibility with athletic performance [48]. In addition, a recent randomized controlled trial demonstrated that pea protein supplementation provided similar improvements in muscle strength and mass as whey protein when combined with resistance training, highlighting its role as a sustainable alternative [78].

Despite these benefits, plant-derived proteins also present challenges. Highly processed proteins, particularly soy isolates, may cause allergic reactions, and nuts, soy, and wheat remain among the most common allergens [66]. Emerging proteins such as peas and green beans lack sufficient allergenicity data, and current sequence databases are limited for robust safety assessments [51]. Furthermore, the presence of anti-nutrients and potential deficiencies in iron, zinc, and vitamin B12 represent additional risks [19]. Nevertheless, plant-derived proteins remain among the most widely available and sustainable alternatives, and continued research and innovation are expected to enhance their nutritional quality and applicability in sports nutrition.

#### Insect Proteins

Edible insects are increasingly considered an alternative to animal meat because of their nutritional value and lower environmental impact [3, 9]. They are commonly consumed in Southeast Asia, Central and West Africa, and Central and South America, while acceptance in Western countries remains low due to cultural taboos, associations with unhygienic conditions, and feelings of disgust [80, 81, 90].

Approximately 1900 insect species are consumed worldwide and are recognized as a valuable source of protein, essential amino acids, and fatty acids [41, 58]. Oils obtained from certain species contain higher amounts of unsaturated fatty acids than meat and generally provide omega-3 fatty acids, which are associated with positive health effects. Insects also offer sustainability advantages such as lower feed costs, reduced environmental pollution, and benefits related to water use, greenhouse gas emissions, waste reduction, animal welfare, feed conversion efficiency, and infection risk [23, 56].

The nutritional composition of edible insects is diverse, including proteins, lipids, chitin, vitamins, and minerals. However, values may differ widely depending on species, developmental stage, diet, and processing methods.

##### • Protein

Protein is the most abundant nutrient, ranging between 20% and 70% of dry matter, with reports up to 80% in some species. Insects provide all essential amino acids, and their digestibility can range from 46% to 96%, comparable to or higher than many plant and animal proteins [59].

##### • Lipids

Lipid content varies from 2% to 50% of dry matter. Fatty acid profiles are generally favorable, dominated by unsaturated fatty acids such as oleic, linoleic, and linolenic acids. Larval and pupal stages tend to have higher fat levels, with some species reaching over 40% lipid content [94].

##### • Chitin

The exoskeleton of insects contains chitin, usually 8–27% of dry weight, which functions as dietary fiber. Chitin not only contributes to structural integrity but also exhibits antioxidant and antimicrobial properties, though it can reduce protein digestibility when strongly bound to muscle proteins [42].

##### • Minerals and Vitamins

Insects are rich in minerals such as calcium, phosphorus, potassium, magnesium, zinc, and iron. For example, cricket powder can cover up to 20% of daily calcium requirements, and the iron bioavailability of some species surpasses that of beef. They are also notable sources of vitamins including riboflavin, thiamin, folic acid, vitamin B12, and vitamin A. Some species contain vitamin B12 levels ten times higher than beef, underlining their potential as a micronutrient-dense food source [44, 59].

Safety concerns regarding the consumption of edible insects may be associated with pathogenic microorganisms in these insects [28]. The European Food Safety Authority (EFSA) has issued scientific opinions indicating that certain insect species are safe as novel foods for human consumption between 2021 and 2023. The European Commission has granted approval for consumption in certain forms based on EFSA’s scientific assessment of the safety of these species. The species and years of authorization are as follows:

##### 1. Migratory locust (*Locusta migratoria*)

In 2021, it was found safe for consumption in frozen, dried, and powdered form.

##### 2. House Cricket (*Acheta domesticus*)

Approved in 2022 for frozen, dried and powdered consumption.

##### 3. Yellow mealworm (*Tenebrio molitor*)

Recognized in 2021 as safe for consumption in dried form.

##### 4. Small Mealworm (*Alphitobius diaperinus*)

Approved in 2023 for consumption in frozen, paste, dried, and powdered form [33].

Although few studies relate insect protein consumption to sports, some show promising results. The study by Zielińska and Pankiewicz examined the nutritional value, micronutrient properties, amino acid profile, and chemical score of insect proteins as alternative protein sources for athletes using protein supplements. In particular, the proteins of the banded grasshopper *(Gryllodes sigillatus)* flour, defatted flour, and protein preparation forms were compared with popular commercial protein supplements. The antioxidant activity of insect protein hydrolysates was higher than that of commercial supplements, particularly in the defatted formula [95]. In a randomized controlled crossover study, Vangsoe et al. [88] compared postprandial amino acid concentrations after ingestion of protein isolates from mealworm, whey, and soy in young men. They observed similar increases in BCAAs and leucine, with insect protein behaving as a more “slowly digestible” protein.

Furthermore, the supplements and eating habits, food neophobia, nutritional knowledge, and willingness to taste edible insects of Italian professional athletes revealed that curiosity about protein content and texture were key drivers of acceptance, with male athletes showing higher approval for insect-based products [64]. Hermans et al. [29] also demonstrated that consuming mealworm protein (30 g) stimulated postprandial muscle protein synthesis both at rest and during recovery from exercise, with results comparable to milk protein. Insect protein isolates are generally considered a high-quality protein source due to their amino acid profile [14]. Recent systematic reviews support these findings: Rutherford et al. [73] confirmed that despite slightly lower postprandial amino acid concentrations compared with whey or milk, insect proteins supported skeletal muscle anabolism at levels comparable to animal proteins.

Therefore, insect proteins represent a promising, sustainable, and digestible alternative protein source for athletes. However, further large-scale, long-term clinical trials are warranted to confirm their efficacy and safety in sports nutrition [45].

#### Mycoproteins

Mycoproteins are a protein source that has recently received increasing attention and may provide health benefits. They are rich in both dietary fiber and non-animal protein, making them a nutritious option [12]. These proteins are obtained from different types of fungi, especially *Fusarium venenatum*. They contain high-quality protein with essential amino acids and have a biological value similar to meat [46].

From an environmental perspective, mycoproteins have a lower carbon footprint than traditional meats and help reduce food waste. They are generally well tolerated and not associated with common allergic reactions, which further supports their role as a sustainable protein source [47]. Nutritionally, mycoproteins are characterized by high biological value, dietary fiber, and various minerals and vitamins, although they are relatively low in B-complex vitamins. They are low in fat and saturated fatty acids but rich in polyunsaturated fatty acids, particularly linoleic and alpha-linolenic acids [1, 16].

Clinical studies have shown that mycoprotein intake can reduce total cholesterol levels, especially in individuals with hyperlipidemia. They are also a promising source of essential amino acids that can stimulate muscle protein synthesis, making them relevant for sports nutrition. In particular, mycoproteins provide longer and more stable hyperinsulinemia and hyperaminoacidemia compared with milk protein. Consuming 70 g of mycoprotein (31.5 g protein) increased the rate of muscle protein synthesis more than 31 g of milk protein (26 g protein), both at rest and after a bout of resistance exercise. Based on these findings, it has been suggested that mycoproteins be included in the diets of individuals who regularly perform resistance training [54].

In another study, the consumption of a BCAA-enriched mycoprotein drink (35 g, containing 18.7 g protein, 2.5 g leucine, 1.5 g isoleucine, and 1.9 g valine) stimulated muscle protein synthesis at rest and after a bout of resistance exercise. Moreover, as intake increased, higher levels of muscle protein synthesis were observed, even though plasma BCAA concentrations changed only slightly, suggesting that mycoprotein itself was responsible for the anabolic effect [55].

In conclusion, mycoproteins represent an essential alternative protein source for a healthy and sustainable diet. Considering their positive effects on human health and the environment, as well as their potential to stimulate muscle protein synthesis, further investigation and application in sports nutrition are warranted.

#### Microalgae

Algae and photosynthetic eukaryotes can be categorized as microalgae and macroalgae. Microalgae represent a diverse group with almost 200,000 species and have been tested for various applications including food additives, cosmetics, animal feed, and wastewater treatment. They are also a promising source for the formulation of meat alternatives due to their high growth rates compared with other plant protein sources. The annual yield of microalgae ranges from 15 to 30 tons per hectare, while soybean yield is approximately 1.5–3.0 tons per hectare [39].

The United States Food and Drug Administration (FDA) has recognized only a limited number of microalgae species as Generally Recognized as Safe (GRAS). These include *Arthrospira maxima*,* Arthrospira platensis*,* Chlamydomonas reinhardtii*,* Chlorella protothecoides*,* Dunaliella bardawil*,* Haematococcus pluvialis*,* Prototheca moriformis*, and *Schizochytrium sp.* In addition, the Dietary Supplements Information Expert Committee (DSI-EC) has identified Spirulina (*Arthrospira platensis* and *Arthrospira maxima*) as a safe dietary supplement [20].

Protein is a major component of microalgal biomass. Species such as *Arthrospira platensis* may contain up to 70% protein in dry weight, while other GRAS species average around 40% [84]. More recent analyses confirm that protein levels can range between 6% and 71% depending on the strain, with *Arthrospira*,* Chlorella*,* Dunaliella*,* Scenedesmus*, and *Chlamydomonas* often exceeding 40%. Importantly, essential amino acid profiles of several species meet FAO/WHO requirements, and *A. platensis* proteins demonstrate ileal digestibility of about 85%, similar to milk proteins [91].

Microalgae also provide a valuable lipid fraction, typically between 7% and 40% of dry weight. Certain species, such as *Nannochloropsis* and *Schizochytrium*, are particularly rich in polyunsaturated fatty acids (PUFAs), especially eicosapentaenoic acid (EPA) and docosahexaenoic acid (DHA), making them attractive alternatives to fish oil as sustainable omega-3 sources [69].

In terms of vitamins and minerals, microalgae supply a broad spectrum including vitamin A, B-complex (notably riboflavin and B6), vitamin D, and vitamin E. For example, Tetraselmis species contain vitamin A levels exceeding those in carrots, while iron from Chlorella has shown higher bioavailability than that from beef. Substantial amounts of zinc, magnesium, and calcium further contribute to their nutritional density [93]. Microalgae are also rich in bioactive pigments with antioxidant properties. Carotenoids such as β-carotene, lutein, and zeaxanthin are abundant, while *Haematococcus pluvialis* is recognized as the main natural source of astaxanthin, a compound reported to exhibit stronger antioxidant activity than vitamin E or tea polyphenols [69, 93].

Despite these advantages, microalgae pose challenges due to unfavorable sensory properties. Their marine or fishy taste and odor reduce consumer acceptability. Therefore, masking these flavors with stronger aromas or combining algae with other protein sources is often necessary. This issue is particularly important in sports nutrition, as relatively large amounts of algae would be required to provide a single serving of protein, making taste and smell a barrier to use. Although smaller quantities of algae can be combined with other proteins, further research and product development are required to enhance their potential as a protein source and functional food ingredient [24].

Several trials have explored the effects of Spirulina supplementation on exercise performance and recovery. In a randomized trial with 90 male athletes, 60 days of Spirulina supplementation (3 g/day) significantly reduced post-exercise malondialdehyde, blood lactate, and recovery heart rate compared with control, with effects comparable to a commercial antioxidant supplement, although no changes were observed in VO₂max or exercise time [36].

In a double-blind randomized crossover study with trained cyclists, 21 days of Spirulina supplementation (6 g/day) lowered heart rate and blood lactate during submaximal cycling, increased hemoglobin concentration, and significantly enhanced power output during repeated sprints, although no improvements were observed in VO₂max or time trial performance [26]. In overweight and obese men, 12 weeks of Spirulina maxima supplementation (4.5 g/day) combined with an exercise program produced synergistic benefits, improving body composition, VO₂max, resting heart rate, onset of blood lactate accumulation, and time to fatigue compared with Spirulina or exercise alone [30]. In contrast, in rugby athletes, daily Spirulina platensis supplementation (5.7 g/day for seven weeks) showed no significant effects on body composition, strength, or aerobic capacity [11].

Beyond Spirulina, experimental studies with seaweed (*Gracilaria asiatica*) demonstrated improved muscle mass, oxidative stress reduction, and enhanced exercise capacity in rodents [38]. Recent reviews summarize that Spirulina may exert its effects through antioxidant activity, enhanced iron bioavailability, and improved hemoglobin synthesis, while Chlorella may contribute via nitric oxide–mediated vasodilation, though findings remain inconsistent across studies [27]. Overall, these findings highlight microalgae, particularly Spirulina and Chlorella, as promising yet still underexplored protein sources in sports nutrition. While current evidence suggests potential benefits for antioxidant defense, vascular health, and exercise performance, further large-scale, well-controlled human trials are required to confirm their efficacy and practical application in athletes.

#### Cultured Meat

Cultured meat is one of the most promising alternatives to conventional meat, as it enables the explantation of stem cells (including endothelial cells, blood cells, and fibroblasts) without raising or slaughtering animals. Meat products can be obtained directly by growing these cells under controlled conditions [10, 13, 76]. Cultured meat is designed to replicate the taste and texture of traditional meat, while providing a more sustainable and environmentally friendly alternative to livestock production. Therefore, it is regarded as a potentially revolutionary food technology [53, 65].

Unlike plant-based protein sources, the nutritional composition of cultured meat is equivalent to that of conventional meat because it is derived directly from muscle cells [82]. Environmental assessments have shown that compared to conventional meat production, cultured meat can reduce energy use by 7–45%, greenhouse gas emissions by 78–96%, land use by 99%, and water use by 82–96%, depending on the product compared [85].

This approach, often referred to as “sacrifice-free meat,” is also supported by animal rights advocates, as no animals are harmed during the production process. It may reduce problems such as allergic reactions and offers a safer product for consumers. Nevertheless, concerns remain regarding its perceived unnatural origin, long-term health effects, production costs, and consumer acceptance. Looking forward, advances in tissue engineering and computer-aided design may allow the 3D printing of cultured meat products with customized nutritional and sensory properties [82]. In summary, cultured meat represents a promising and innovative technology that combines nutritional equivalence to traditional meat with substantial environmental and ethical benefits, but its large-scale adoption will ultimately depend on overcoming technical barriers, production costs, and consumer acceptance [53]. These aspects are summarized in Table 1.

**Table 1:** Comparison of conventional and cultured meat in terms of nutrition, environmental impact, ethics, and challenges (adapted from [53, 65, 82, 85]

Aspect	Conventional Meat	Cultured Meat
Nutrition	High-quality protein, essential amino acids, vitamins (B12, iron, zinc).	Equivalent protein quality, potential for nutrient tailoring (e.g., fatty acid profile).
Environment	High GHG emissions, land use, water consumption.	7–45% less energy, 78–96% less GHG, 99% less land, 82–96% less water.
Ethics/Animal Welfare	Requires raising and slaughtering animals.	“Sacrifice-free meat,” animal welfare friendly, supported by animal rights advocates.
Challenges	Established production, widely accepted by consumers.	High production costs, consumer acceptance issues, regulatory hurdles, “unnatural” perception.

## Conclusion

The increasing demand for alternative protein sources that align with environmental sustainability goals is particularly relevant in sports nutrition, where protein requirements are higher than in the general population. Alternative protein sources examined in this study—including plant proteins, edible insects, mycoproteins, microalgae, and cultured meat—offer promising solutions that combine nutritional adequacy with reduced environmental impact.

Insect proteins can be considered an ideal protein source for athletes, as they provide all essential amino acids with high digestibility and a low environmental footprint. Mycoproteins also represent a valuable option with high-quality protein and fiber content, although further technological improvements and strategies to increase consumer acceptance are needed to support their widespread use. Microalgae attract attention with their high protein content and rich essential amino acid profiles, yet their application in food products remains limited due to sensory challenges. Research on cultured meat is still at an early stage, with limited studies assessing its nutritional and physiological effects in humans.

Overall, while alternative proteins have considerable potential as natural sources of biologically active compounds, the current body of evidence is largely limited to in vitro studies. Therefore, further research in animal models and well-controlled human trials is necessary to evaluate their efficacy, safety, and long-term health impacts. Integrating these alternative protein sources into sports nutrition could significantly reduce the environmental footprint of protein production while addressing the high nutritional demands of athletes. However, successful adoption will depend not only on technological advances but also on consumer awareness and acceptance, which should be prioritized alongside product development.

## Data Availability

No datasets were generated or analysed during the current study.
